# Pulmonary melioidosis in Cambodia: A prospective study

**DOI:** 10.1186/1471-2334-11-126

**Published:** 2011-05-14

**Authors:** Blandine Rammaert, Julien Beauté, Laurence Borand, Sopheak Hem, Philippe Buchy, Sophie Goyet, Rob Overtoom, Cécile Angebault, Vantha Te, Patrich Lorn Try, Charles Mayaud, Sirenda Vong, Bertrand Guillard

**Affiliations:** 1 Institut Pasteur - Cambodia, Phnom Penh, Cambodia; 2 Swiss Red Cross, Takeo, Cambodia; 3 Donkeo Provincial Hospital, Takeo, Cambodia; 4 Kampong Cham Provincial hospital, Kampong Cham, Cambodia; 5 Centre de pneumologie et réanimation respiratoire, Hôpital Tenon, Paris, France

**Keywords:** *Burkholderia pseudomallei*, community-acquired pneumonia, tuberculosis, Cambodia

## Abstract

**Background:**

Melioidosis is a disease caused by *Burkholderia pseudomallei *and considered endemic in South-East Asia but remains poorly documented in Cambodia. We report the first series of hospitalized pulmonary melioidosis cases identified in Cambodia describing clinical characteristics and outcomes.

**Methods:**

We characterized cases of acute lower respiratory infections (ALRI) that were identified through surveillance in two provincial hospitals. Severity was defined by systolic blood pressure, cardiac frequency, respiratory rate, oxygen saturation and body temperature. *B. pseudomallei *was detected in sputum or blood cultures and confirmed by API20NE gallery. We followed up these cases between 6 months and 2 years after hospital discharge to assess the cost-of-illness and long-term outcome.

**Results:**

During April 2007 - January 2010, 39 ALRI cases had melioidosis, of which three aged ≤2 years; the median age was 46 years and 56.4% were males. A close contact with soil and water was identified in 30 patients (76.9%). Pneumonia was the main radiological feature (82.3%). Eleven patients were severe cases. Twenty-four (61.5%) patients died including 13 who died within 61 days after discharge. Of the deceased, 23 did not receive any antibiotics effective against *B. pseudomallei*. Effective drugs that were available did not include ceftazidime. Mean total illness-related costs was of US$65 (range $25-$5000). Almost two-thirds (61.5%) incurred debt and 28.2% sold land or other belongings to pay illness-related costs.

**Conclusions:**

The observed high fatality rate is likely explained by the lack or limited access to efficient antibiotics and under-recognition of the disease among clinicians, which led to inappropriate therapy.

## Background

Melioidosis is an infectious disease due to non-saprophytic Gram-negative bacillus *Burkholderia pseudomallei*, which can be found in wet soil and surface water [1]. Endemic areas encompass Southeast Asia and Northern Australia demonstrating seasonality with more cases detected/reported during the rainy season [2,3]. In addition to contact with an environmental exposure, underlying conditions pre-disposing for the disease include diabetes mellitus, alcohol abuse, chronic lung disease and chronic renal disease [1,4]. The three main routes of contamination are cutaneous inoculation, inhalation and ingestion [5]. Presentation of the disease is highly variable ranging from acute septicemia to chronic localized abscess while most infections with *B. pseudomallei *are asymptomatic [6]. Lungs are the most commonly infected organs but every organ may be affected. Other frequent localizations include skin abscess, osteomyelitis, arthritis, prostatic and parotids abscesses [1,7,8]. Diagnosis of melioidosis relies on the isolation of *B. pseudomallei *in clinical samples (mostly blood, sputum and pus). Serology is useful for epidemiological surveys but of little diagnostic interest since most of the population is sero-positive in endemic areas [9]. Analysis of resistance patterns of the *B. pseudomallei *strains showed that severe cases of melioidosis were best treated with at least 10 days of intravenous intensive therapy with ceftazidime followed by 12 to 20 weeks of an oral eradication therapy using a three-drug regimen (i.e. trimethoprim-sulfamethoxazole and doxycycline) [5,10]. Outcome depends on both severity of cases and the level of health services. Septicemia and acute pulmonary forms are the most severe with fatality rates as high as 40%, despite appropriate treatment [1].

Melioidosis remains poorly documented in many countries of the endemic areas [4]. This applies to Cambodia where first indigenous cases were recently reported [11-13]. A study in Siem Reap province reported the detection of antibodies against *B. pseudomallei *in 16% of Cambodian children and isolation in 30% of the surrounding rice fields, which indicates high exposure to the pathogen [14].

This report summarizes the characteristics of pulmonary melioidosis cases among patients hospitalized with acute lower respiratory infection and the results of a follow-up investigation of these cases several months after hospital discharge.

## Methods

In April 2007, we implemented surveillance of acute lower respiratory infections (ALRI) in two Cambodian provincial hospitals (Takeo, Southern province and Kampong Cham, Eastern province) combined with identification of bacterial and viral etiologies. Four adult (Internal Medicine, Infectious Disease, Intensive Care) and two Pediatric wards participated in the surveillance study. Eligibility criteria for ALRI included symptoms onset ≤14 days, fever ≥ 38°C or a history of febrile episodes within the previous 3 days, recent cough, plus at least one of the following respiratory symptoms: dyspnea, chest pain or crackles on lung auscultation. Patients with known tuberculosis (TB), or known acquired immunodeficiency such as HIV, chronic corticosteroids use and cancer were excluded.

For each participant, hospital clinicians were asked to complete a standardized surveillance form, including information on patient's medical history, clinical features, treatment, laboratory and radiological findings and patients' outcome at discharge. A chest X-ray was requested for each enrolled patient within 24 hours from admission; blood tests (i.e. complete blood cells count, glycemia, liver enzymes, blood creatinine levels and serum electrolytes) and sputum smear microscopy for acid-fast bacilli detection were performed on site. The surveillance forms and chest X-rays were retrospectively reviewed by an expert pulmonologist.

Blood and sputum were sent daily to the *Institut Pasteur *- Cambodia (IPC) for culture and antibiotic susceptibility testing (AST). *B. pseudomallei *was cultured from sputum on Drigalski and Ashdown's agar and identified by API20NE gallery (BioMérieux). Blood sample was taken on Hemoline Diphasic Performance bottle (BioMérieux) for adults and Isolator 1.5 (Oxoid) (1.5 ml) for children. We determined antimicrobial drug susceptibility by the disk-diffusion method on Mueller-Hinton agar plates (Bio-Rad). When *B. pseudomallei *appeared resistant to cotrimoxazole, Minimum inhibitory concentrations were determined using the E-test diffusion method (AB Biodisk) to confirm or infirm resistance [1]. Bacteriology results were returned to the patient's clinicians as soon as they were available, together with AST.

### The follow-up investigation

For the purpose of the study, we visited and interviewed all confirmed melioidosis cases at home within six months after hospital discharge to assess the patient's status and collect additional information using a standardized questionnaire. We collected information regarding known risk factors for melioidosis (i.e. previous pulmonary disease or history of smoking, diabetes mellitus, chronic disease, alcohol intake, repeated contact with wet soils), medical history of infection, wounds or abscesses, time to diagnosis, disease evolution, date of death if appropriate, and economic burden on patients' families. Patients were visited a second time between 6 months and 2 years after the first home visit to follow-up on possible relapse.

### Definitions

A pulmonary melioidosis case was defined as a patient with a febrile illness and respiratory symptoms with the identification of *B. pseudomallei *in sputum and/or blood culture. A severe case was defined by the presence of at least two of the following criteria: systolic blood pressure <90 mmHg, cardiac frequency ≥120 beats-per-minute, respiratory rate ≥30/mn, oxygen saturation <90%, temperature <35°C or ≥40°C. For children, severity was assessed using the World Health Organization criteria [15]. In the present study, we defined chronic forms of melioidosis when re-assessment (follow-up investigation) of patient's medical history revealed onset of symptoms >14 days. Since no patient was taking anti-diabetic drug, we retained as diabetic every patient with glycemia >126 mg/dL or a history of diabetes mellitus. Chronic lung disease includes chronic bronchitis and pre-existing pulmonary lesions. Renal impairment was defined by serum creatinine levels > 2 mg/dL. A necrotizing pneumonia was defined by the presence of radiographic imaging of either cavity or abscess, unique or multiple.

### Treatment

Antibiotics that are effective against *B. pseudomallei *and available in Takeo and Kampong Cham include sulfamethoxazol-trimethoprime (cotrimoxazole), chloramphenicol, doxycycline, co-amoxiclav. The standard treatment protocol was based on the Thai guidelines, 22 weeks of a four-antibiotic regimen including cotrimoxazole, chloramphenicol and doxycycline. If the patient's condition had significantly improved on week 8 of treatment, chloramphenicol would be discontinued [1]. An out-patient follow-up was implemented at each hospital during and after the treatment.

### Ethics

The surveillance study was approved by the Cambodian National Ethical Committee. All patients/parents of sick children who participated in surveillance provided written informed consent.

### Statistical methods

Statistical analyses were performed using Stata version 9.0 (Statacorp, College Station, TX, USA). Continuous variables were presented with their median (minimum and maximum ranges) or with their mean and standard deviation. Variables were compared across groups using the Wilcoxon-Mann-Whitney test for continuous variables, and the Chi-square or Fisher's exact test for categorical variables. For all analyses, statistical significance was defined as *P *< 0.05.

## Results

### Patients' characteristics

From April 2007 to February 2010, 2,840 patients with ALRI (i.e. pneumonia, abscess, pleural effusion, superinfection on lung sequelae, bronchitis) were reported through surveillance and 2,407 had at least one sample available (1,985 in Takeo and 422 in Kampong Cham) for bacteriological testing. Among these patients with ALRI, 39 (1.6%) were confirmed as having been infected by *B. pseudomallei*, of which 35 were treated at Takeo and 4 at Kampong Cham hospital; the proportion of *B. pseudomallei *isolates among positive cultures was 9.3% including 51.4% in blood culture and 7.7% in sputum culture. The demographics and baseline characteristics of patients are presented in Table 1. The median age on admission was 46 years (range 1 month - 74 years). Among the 39 patients, 3 were children of which one was less than two months old. Two adults had a history of near-drowning. Underlying conditions identified as a risk factor were reported in 23 (59.0%) patients (Table 1). Among these 23 patients, 7 had diabetes mellitus and one tested positive for HIV antibodies. A soil exposure or close contact with water was identified in 30 (76.9%) patients. All of them reported working in rice fields and some reported additional activities such as fishing, collecting water plants or working in construction. One patient was pregnant, but her pregnancy term could not be ascertained.

**Table 1:** General characteristics of the 39 melioidosis cases

	All patients (n = 39)	Non severe (n = 28)	Severe (n = 11)	P value
Male	22 (56.4%)	19 (67.9%)	3 (27.3%)	0.03
Age, median years (min. - max.)	46 (0.08-74)	47.5 (19-72)	29 (0.08-74)	
Risk factors				
Close contact with wet soil	30 (76.9%)	23 (82.1%)	7 (63.6%)	
Underlying chronic disease	23 (59.0%)	21 (75.0%)	2 (18.2%)	<0.01
Diabetes mellitus	7 (18.0%)	7 (25.0%)		
Renal impairment	4 (10.3%)	3 (10.7%)	1 (9.1%)	
Chronic lung disease	13 (33.3%)	12 (42.6%)	1 (9.1%)	
Alcoholism	12 (30.8%)	10 (35.7%)	2 (22.2%)	
Data on admission				
Temperature ≥ 38°C	27 (69.2%)	17 (60.7%)	10 (90.9%)	
Cough	38 (97.4%)	27 (96.4%)	11 (100%)	
Dyspnea	30 (76.9%)	21 (75.0%)	9 (81.8%)	
Thoracic pain	31 (79.5%)	26 (92.9%)	5 (45.5%)	<0.01
Hemoptysis	1 (2.6%)	1 (3.7%)		
Leucocytes median 10^3^/mm^3^	9.4 (6.5-14.4)	10.9 (7.6-16.6)	6.4 (3.2-9.4)	<0.01
Pneumonia	27/34 (79.4)	20/27 (74.0)	7/7 (100)	
Pleural effusion	2/28 (7.1%)	2/27 (7.4%)		
Outcome				
Death	24/39 (61.5%)	13/15 (46.4%)	11/11 (100)	<0.01
Time to death, days (min-max)	3 (1-61)	3 (1-61)	3 (1-14)	

### Clinical and microbiological diagnosis

Of the reported cases, 15 were confirmed by sputum culture, 18 by blood culture and 6 by both. In-depth interviews during the follow-up investigation actually identified 15 (38.5%) cases that had symptoms onset beyond 14 days (median 30 days, range 21 days - 3 years); onset of symptoms could not be ascertained for four patients. Eleven (28.2%) patients including all pediatric cases were severe. None of the two patients with history of near drowning was severe on admission. Twenty-eight (71.8%) were identified during the rainy season (from May to October). Detailed clinical and laboratory data are presented in Table 1. Productive cough was present in 28 (71.8%) cases. One patient had hemoptysis without cavity on chest radiography. Leucocytes count was significantly higher in acute melioidosis (14.3% vs. 8.5%; p < 0.01) and in non-severe cases (10.9% vs. 6.4%; p < 0.01). Liver enzymes level did not significantly differ between severe/non-severe cases or acute/chronic forms.

A positive blood culture was significantly associated with severe cases (10/11 vs. 14/26; p = 0.03). Although 5/7 (71.4%) diabetic patients had positive blood culture, none was severe. No patient had bacterial co-infection; however, two of the 3 children had H3N2 *influenza A *virus infection. Bacteriological results and *B. pseudomallei *sensitivity to main antibiotics are displayed in Tables 2 and 3. All the strains were susceptible to cotrimoxazole with E-test method, although 20 (51.3%) were not susceptible with disk-diffusion method.

**Table 2:** Types of bacteriological results from melioidosis cases

	All patients (n = 39)	Non severe (n = 28)	Severe (n = 11)	P value
Source of isolates				
Sputum	21/22 (95.5%)	19/20 (95.0%)	2/2 (100%)	
Blood culture	24/37 (64.9%)	14/26 (53.8%)	10/11 (90.9%)	0.03
Sputum and blood culture	6 (15.4%)	5 (17.9%)	1 (9.1%)	
Resistant strains to co-amoxiclav	5 (13.5%)	3 (11.5%)	2 (18.2%)	

**Table 3:** In vitro activities of selected antibiotics against 39 strains of *B. pseudomallei*

Agent	Sensitive	Intermediate	Resistant
Amoxicillin-clavulanate	32 (82.1%)	2 (5.1%)	5 (12.8%)
Aztreonam	29 (74.4%)	10 (25.6%)	
Ceftazidime	39 (100%)		
Chloramphenicol	38 (97.4%)		1 (2.6%)
Cotrimoxazole*	39 (100%)		
Imipenem	39 (100%)		
Piperacillin	39 (100%)	1 (2.6%)	
Piperacillin-tazobactam	39 (100%)	1 (2.6%)	
Tetracyclin	38 (94.8%)	1 (2.6%)	
Ticarcillin		4 (10.3%)	35 (89.7%)
Ticarcillin-clavulanate	12 (30.8%)	15 (38.4%)	12 (30.8%)

*Susceptibility testing was done by diffusion disk method for all antibiotics. E-test method was only used to detect resistance to cotrimoxazole

### Radiological features

**Figure 1:**
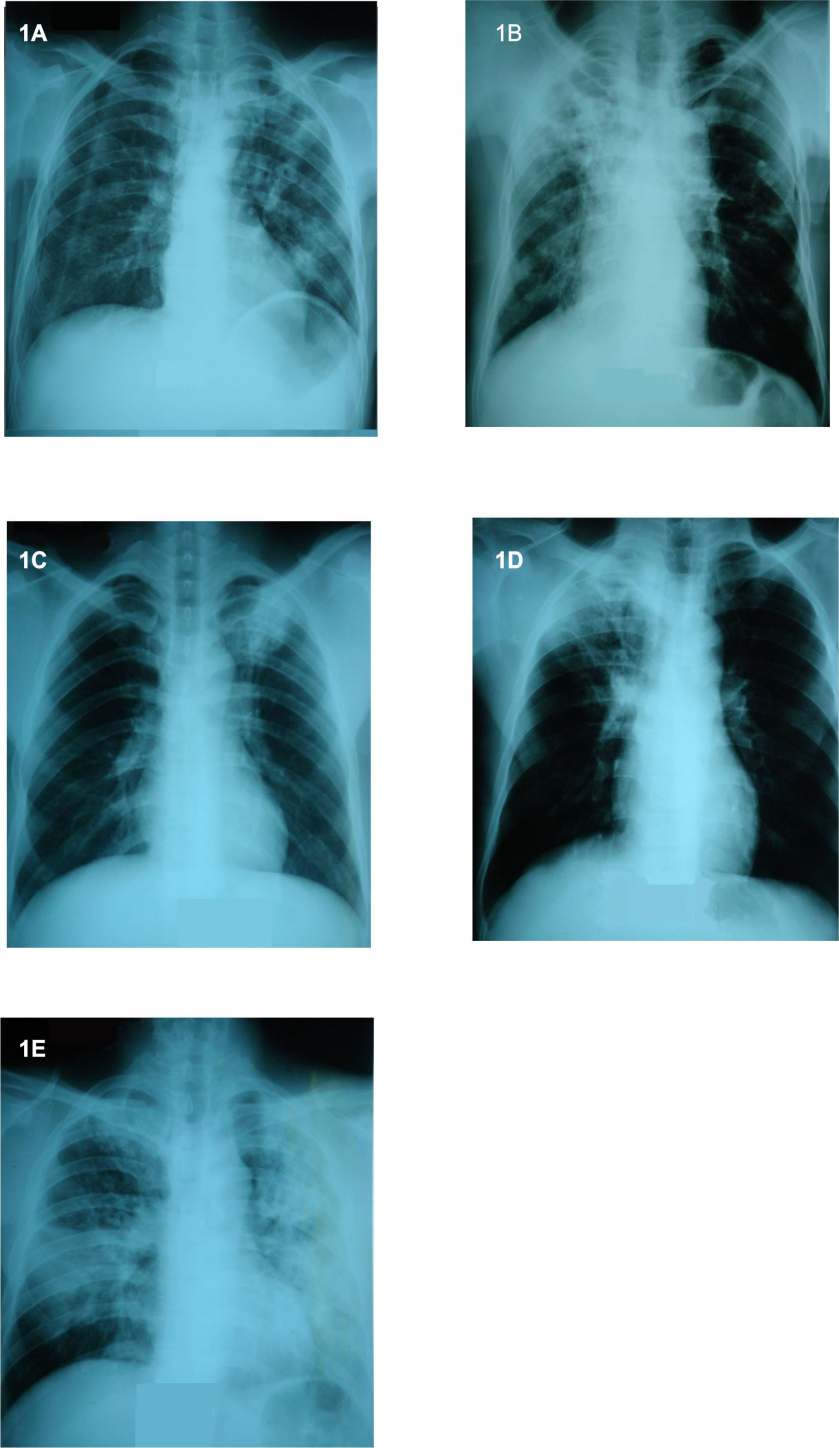
**Chest radiographs evoking tuberculosis in 5 patients with pulmonary melioidosis**. A: 48 year-old male; B: 52 year-old male; C: 52 year-old male who had a positive smear for acid-fast bacilli; D, 36 year-old male; E, 43 year-old male

Chest radiographs were only available in 34 (87.2%) cases (Table 4). Of these, 5 patients had no evidence of pneumonia or pleural effusion, 2 only had pleural effusions and 27 (79.4%) had pneumonia associated with abscesses, nodules or pleural effusions; 16 (59.3%) pneumonia consisted of pulmonary lesions in two or more lobes. All severe melioidosis cases for which chest radiographs were available showed evidence of pneumonia with either alveolar consolidation (n = 2), nodules (n = 3) or pleuropneumonia (n = 2). Two patients who had normal chest radiographs tested positive for *B. pseudomallei *by blood culture. Pulmonary sequelae were found on 3 chest radiographs including emphysema, probable TB sequelae and bronchiectasis related- post-infection. Interestingly the experts panel interpreted chest radiographs of five cases of melioidosis as TB-like (Figure 1); all but one were negative for acid-fast bacilli (AFB) by direct sputum examination (in line with the national TB program, AFB negative pneumonias evocative of TB are not systematically cultured). Three of these five cases had chronic melioidosis.

**Table 4:** Radiographic features on acute and and chronic melioidosis cases' chest X-rays

	All patients (n = 34*)	Patients with acute melioidosis (n = 19)	Patients with chronic melioidosis (n = 13)
Pneumonia**	27 (79.4%)	13 (68.4%)	12 (92.3%)
Necrotizing	10 (29.4%)	6 (31.6%)	4 (30.8%)
Nodular	5 (17.9%)	1 (5.3%)	2 (15.4%)
With pleural effusion	4 (11.8%)	1 (5.3%)	3 (23.1%)
Pleural effusion	2 (5.9%)	1 (5.3%)	1 (7.7%)
No pneumonia or pleural effusion	5 (14.7%)	5 (26.3%)	
Pulmonary sequelae	3 (8.8%)	3 (15.8%)	
Normal X-ray	2 (5.9%)	2 (5.9%)	

* 5/39 patients did not have an X-ray of which 4 were severe on admission; onset of symptoms could not be documented in 2/34 patients, ** P > 0.05 between acute and chronic melioidosis

### Treatment and outcome

Only 13 patients (none were severe) received antibiotics active against *B. pseudomallei *either during hospitalization or after discharge. The mean duration of hospitalisation was 6 days (range 1 - 11 days) with a shorter duration for severe patients (2 days [1-4] vs 8 [2-12]; p < 0.01). Overall fatality rate was of 61.5% (24/39); 45.8% (11/24) died during admission. Among the deceased, 23 (96%) patients died without having received any of the active drugs (i.e. cotrimoxazole, chloramphenicol, doxycycline, amoxicillin-clavulanate acid). The pregnant woman died 5 days after discharge; all pediatric cases died at hospital. The 2 month-old child's mother could not be found to assess the potential routes of transmission. The median time from hospital admission to death was 3 days (range 1 - 61 days). Fatality was significantly higher in severe cases (100% (11/11) vs. 46.4% (13/28); p < 0.01). There were no differences in fatality or severity between acute and chronic cases. Positive blood culture (21/24 (87.5%) vs. 3/15 (20%); p < 0.01) and none active drug prescribed (23/26 (88.5%) vs. 1/13 (7.7%); p < 0.001) were also significantly associated with a higher fatality.

### Follow-up visits

Of the 28 patients who were discharged, 24 could be located and visited; only 11 (45.8%) remained alive. Of these 11, 8 (72.7%) attended follow-up consultations as recommended. Nine received a treatment with at least 2 active antibiotics after hospital discharge, of which seven interrupted antibiotics treatment complaining about the side effects or lack of money. Nevertheless all improved and were considered as cured during the second follow-up visit (median 567 days after discharge, range 420 - 1028). Chest X-rays were done at the time of the second visit in 9 patients who were initially admitted with radiographic evidence of pneumonia or pleural effusions. The results showed radiographic recovery (normal chest X-ray) in four patients while the remaining five had radiographic imaging of lung sequelae.

### Costs

The mean reported cost of the disease was 2,261,378 Riels (US$565; range $25-$5000) including hospital costs, treatment costs and other related costs, such as food or transportation. Almost two thirds of the patients (61.5%, n = 24/39) had to incur debt (range US$150-1000); a third (28.2%, n = 11/39) sold a cow, buffalo, pig or poultries and a quarter (25.6%, n = 10/39) other belongings (gold or land).

## Discussion

This report describes patients with pulmonary melioidosis, detected through surveillance of ALRI. To our knowledge, this is the first report of pulmonary melioidosis cases that were followed several months after hospital discharge in Cambodia [12-14].

Fatality rates (~62%) in our cohort were high compared to that of Thailand (40.5% in 2006) [2,16]. These high rates likely were related to multiple factors, including inadequate antibiotic therapy, low awareness of the disease and limited access to health care services rather than a severity of the disease that is peculiar to Cambodia.

Indeed, awareness of melioidosis among health professionals remains low. In the context of high TB endemicity and low microbiological capacity in public hospitals, misdiagnosis of melioidosis taken as TB may not be uncommon [13]. All the more when six patients' chest X-rays were interpreted as TB-like infection by our clinical experts. In the surveillance study, many clinicians did not account for melioidosis in their first line antibiotic treatment when diagnosing severe pneumonia. Moreover, many patients were discharged before having received bacteriological results; it is recognized in Cambodia that many patients' families would rather go back home if they cannot afford to stay longer at the hospital or when patients are severely ill with little hope of cure.

The turning point in the treatment of bacteremic melioidosis was the use of ceftazidime reducing by half fatality [1,17]. Parenteral co-amoxiclav would have been a cheaper alternative in our study hospital; unfortunately, both drugs are costly and not available in the National Essential Drugs list. With a potentially high burden of melioidosis in Cambodia (12 - 14), one should consider making ceftazidime available by using a generic form that is now manufactured in Thailand (http://www.drugs.com/international/cef-4.html). Notably, oral treatment can still rely on cotrimoxazole - a cheap and available drug - as susceptibility testing for this antibiotic using E-test method has demonstrated no resistance of *B. pseudomallei *isolates. This finding contrasts with that of Northern Thailand where susceptibility of isolates was lower at 87% [18].

The present study showed that melioidosis had a substantial financial impact on households in rural Cambodia. To pay for these costs, two-thirds of households had to borrow money and 25% had to sell assets or use their savings, a finding that is consistent with studies on cost-of-illness for other infectious diseases in Cambodia [19]. To put in perspective, the average cost of melioidosis was similar to the Gross National Income per capita in Cambodia, i.e. US$600 in 2008 [20]. These costs were overwhelming when compared to an average one-week expenditure on food of US$ 9.5 per household [19].

Previous series reported 37 to 60% of diabetes mellitus in patients with melioidosis [5]. Only one study has documented a high prevalence of diabetes mellitus (~61%) in the sub-group of pulmonary melioidosis [16]. Our results were consistent with that of studies which reported higher prevalence of diabetes mellitus in bacteremic patients [21-23]. However, the proportion of diabetes mellitus in our series appeared low. This is surprising as the prevalence of diabetes mellitus in Cambodia is thought to be high, accounting for 11% of the population in Kampong Cham province [24]. Similarly, we observed that severe patients were less likely to have co-morbidities. Diabetes mellitus may have been under-reported among our patients. Since most of these patients died quickly after their admission, it is possible that clinicians were less demanding when interrogating the family on diabetes as an underlying condition.

Our study has additional limitations. Estimating the extent of melioidosis and its severity in ALRI is problematic due to the difficulties in obtaining microbiological diagnoses. Few of our patients were children in whom sputum was difficult to collect and therefore hardly available. However, it is very likely that fewer children suffer from pulmonary melioidosis [5,12]. In Thailand, higher incidence rates were found in 40-60 year old adults [21]. In addition, blood culture is rarely positive in children since they suffer from less severe forms of melioidosis [5]. Of note, one patient was 2 months old at the time of diagnosis suggesting possible vertical transmission from mother to child or a neonatal infection [25]. Unfortunately, we could not trace this child for the follow-up investigation. Two of the three children died and were co-infected by H3N2 influenza virus. It is difficult indeed to relate the severity of the disease to influenza infection, although reactivation of melioidosis following influenza infection has been described in adults [26].

Second, the surveillance study is limited to hospitalized cases of pulmonary infection and so does not include sub-acute forms and other presentations of the disease; the follow-up questionnaire revealed that one third of the patients actually had chronic melioidosis which resulted in overestimating the proportion of melioidosis in severe acute respiratory infections. Nevertheless, we believe that our series is reasonably representative of people in the provinces as our hospitals were the only reference hospitals for their respective provinces.

## Conclusions

We suggest, therefore, that pulmonary melioidosis should be considered in every patient with TB-like chest X-rays and negative AFB smears in Cambodia. The study was able to highlight difficulties faced by Cambodian clinicians when managing and treating melioidosis cases. In addition, it is imperative that Cambodian health professionals understand melioidosis and consider the disease in their differential diagnoses, especially for pulmonary tuberculosis and almost all other febrile infections, particularly during the rainy season. In the meantime, detecting *B. pseudomallei *in an endemic area could actually be implemented with the use of throat swabs. This method with 100% specificity (but low sensitivity, 36%) could be easily disseminated to Cambodian hospitals [27]. Furthermore, strengthening hospital laboratories throughout Cambodia with an easy-to-make Ashdown's culture medium could be easily implemented [28]. These two recommendations taken together would provide further information on the burden of melioidosis in the different provinces in Cambodia and subsequently help advocate for the adoption of a national treatment protocol including ceftazidime.

## Competing interests

The author declares that they have no competing interests.

## Authors' contributions

CA, SV, BG, LB, RO, PB, BR and CM contributed to the design of the study, conducted the study, participated in statistical analysis and interpretation of data, and manuscript preparation. VT, PLT, SG, SH, CA, BR, RO gathered the secondary and collected the data from the field. BR, JB, LB analyzed the data and wrote the first draft of the manuscript. SV, CA and BG conceived the study. SV and BG as senior authors contributed equally to this study and approved the final version of the manuscript. All authors read and approved the final manuscript.

## Pre-publication history

The pre-publication history for this paper can be accessed here:

http://www.biomedcentral.com/1471-2334/11/126/prepub

## References

[B1] White NJ Melioidosis Lancet 2003 361 1715 1722 10.1016/S0140-6736(03)13374-0 12767750

[B2] Limmathurotsakul D Wongratanacheewin S Teerawattanasook N Wongsuvan G Chaisuksant S Chetchotisakd P Chaowagul W Day NP Peacock SJ Increasing incidence of human melioidosis in Northeast Thailand Am J Trop Med Hyg 2010 82 1113 7 10.4269/ajtmh.2010.10-0038 20519609 PMC2877420

[B3] Currie BJ Ward L Cheng AC The epidemiology and clinical spectrum of melioidosis: 540 cases from the 20 year darwin prospective study PLoS Negl Trop Dis 2010 4 e900 10.1371/journal.pntd.0000900 21152057 PMC2994918

[B4] Dance DAB Melioidosis Curr Opin Infect Dis 2002 15 127 132 10.1097/00001432-200204000-00005 11964912

[B5] Cheng AC Currie BJ Melioidosis: epidemiology, pathophysiology, and management Clin Microbiol Rev 2005 18 383 416 10.1128/CMR.18.2.383-416.2005 15831829 PMC1082802

[B6] Kanaphun P Thirawattanasuk N Suputtamongkol Y Naigowit P Dance DA Smith MD White N Serology and carriage of Pseudomonas pseudomallei: a prospective study in 1000 hospitalized children in northeast Thailand J Infect Dis 1993 167 230 233 10.1093/infdis/167.1.230 7678106

[B7] Currie BJ Fisher DA Howard DM Burrow JN Lo D Selva-Nayagam S Anstey NM Huffam SE Snelling PL Marks PJ Stephens DP Lum GD Jacups SP Krause VL Endemic melioidosis in tropical northern Australia: a 10-year prospective study and review of the literature Clin Infect Dis 2000 31 981 986 10.1086/318116 11049780

[B8] Dance DA Melioidosis as an emerging global problem Acta Trop 2000 74 115 119 10.1016/S0001-706X(99)00059-5 10674638

[B9] Wuthiekanun V Chierakul W Langa S Chaowagul W Panpitpat C Saipan P Thoujaikong T Day NP Peacock SJ Development of antibodies to Burkholderia pseudomallei during childhood in melioidosis-endemic northeast Thailand Am J Trop Med Hyg 2006 74 1074 1075 16760522

[B10] White NJ Dance DA Chaowagul W Wattanagoon Y Wuthiekanun V Pitakwatchara N Halving of mortality of severe melioidosis by ceftazidime Lancet 1989 2 697 701 2570956 10.1016/s0140-6736(89)90768-x

[B11] Chan KPW Low JGH Raghuram J Fook-Chong SMC Kurup A Clinical characteristics and outcome of severe melioidosis requiring intensive care Chest 2005 128 3674 3678 10.1378/chest.128.5.3674 16304330

[B12] Pagnarith Y Kumar V Thaipadungpanit J Wuthiekanun V Amornchai P Sin L Day NP Peacock SJ Emergence of pediatric melioidosis in Siem Reap, Cambodia Am J Trop Med Hyg 2010 82 1106 1112 10.4269/ajtmh.2010.10-0030 20519608 PMC2877419

[B13] Overtoom R Khieu V Hem S Cavailler P Te V Chan S Lau P Guillard B Vong S A first report of pulmonary melioidosis in Cambodia Trans R Soc Trop Med Hyg 2008 102 S21 25 19121680 10.1016/S0035-9203(08)70007-5

[B14] Wuthiekanun V Pheaktra N Putchhat H Sin L Sen B Kumar V Langla S Peacock SJ Day NP Burkholderia pseudomallei antibodies in children, Cambodia Emerging Infect Dis 2008 14 301 303 10.3201/eid1402.070811 18258125 PMC2600196

[B15] World Health Organization Pocket book of Hospital Care for Children. Guidelines for the management of common illenesses with limited ressources 2006 World Health Organization. Geneva

[B16] Mukhopadhyay A Lee KH Tambyah PA Bacteraemic melioidosis pneumonia: impact on outcome, clinical and radiological features J Infect 2004 48 334 338 10.1016/j.jinf.2003.10.005 15066335

[B17] Simpson AJ Suputtamongkol Y Smith MD Angus BJ Rajanuwong A Wuthiekanun V Howe PA Walsh AL Chaowagul W White NJ Comparison of imipenem and ceftazidime as therapy for severe melioidosis Clin Infect Dis 1999 29 381 7 10.1086/520219 10476746

[B18] Wuthiekanun V Cheng AC Chierakul W Amornchai P Limmathurotsakul D Chaowagul W Simpson AJ Short JM Wongsuvan G Maharjan B White NJ Peacock SJ Trimethoprim/sulfamethoxazole resistance in clinical isolates of Burkholderia pseudomallei J Antimicrob Chemother 2005 55 1029 31 10.1093/jac/dki151 15886263

[B19] Huy R Wichmann O Beatty M Ngan C Duong S Margolis HS Vong S Cost of dengue and other febrile illnesses to households in rural Cambodia: a prospective community-based case-control study BMC Public Health 2009 9 155 10.1186/1471-2458-9-155 19473500 PMC2696434

[B20] Cambodia data of the World Bank http://data.worldbank.org/country/cambodia [Accessed June 28, 2010].

[B21] Suputtamongkol Y Chaowagul W Chetchotisakd P Lertpatanasuwun N Intaranongpai S Ruchutrakool T Budhsarawong D Mootsikapun P Wuthiekanun V Teerawatasook N Lulitanond A Risk factors for melioidosis and bacteremic melioidosis Clin Infect Dis 1999 29 408 413 10.1086/520223 10476750

[B22] Chaowagul W White NJ Dance DA Wattanagoon Y Naigowit P Davis TM Looareesuwan S Pitakwatchara N Melioidosis: a major cause of community-acquired septicemia in northeastern Thailand J Infect Dis 1989 159 890 899 10.1093/infdis/159.5.890 2708842

[B23] Puthucheary SD Parasakthi N Lee MK Septicaemic melioidosis: a review of 50 cases from Malaysia Trans R Soc Trop Med Hyg 1992 86 683 685 10.1016/0035-9203(92)90191-E 1287945

[B24] King H Keuky L Seng S Khun T Roglic G Pinget M Diabetes and associated disorders in Cambodia: two epidemiological surveys Lancet 2005 366 1633 1639 10.1016/S0140-6736(05)67662-3 16271644

[B25] Abbink FC Orendi JM de Beaufort AJ Mother-to-child transmission of Burkholderia pseudomallei N Engl J Med 2001 344 1171 1172 10.1056/NEJM200104123441516 11302149

[B26] Mackowiak PA Smith JW Septicemic melioidosis. Occurrence following acute influenza A six years after exposure in Vietnam JAMA 1978 240 764 766 10.1001/jama.240.8.764 671709

[B27] Wuthiekanun V Suputtamongkol Y Simpson AJ Kanaphun P White NJ Value of throat swab in diagnosis of melioidosis J Clin Microbiol 2001 39 3801 3802 10.1128/JCM.39.10.3801-3802.2001 11574624 PMC88440

[B28] Walsh AL Wuthiekanun V The laboratory diagnosis of melioidosis Br J Biomed Sci 1996 53 249 53 9069100

